# The adhesion of clots in wounds contributes to hemostasis and can be enhanced by coagulation factor XIII

**DOI:** 10.1038/s41598-020-76782-z

**Published:** 2020-11-18

**Authors:** Karen Y. T. Chan, Alyssa S. M. Yong, Xu Wang, Kristyn M. Ringgold, Alexander E. St. John, James R. Baylis, Nathan J. White, Christian J. Kastrup

**Affiliations:** 1 Michael Smith Laboratories, University of British Columbia, 2185 East Mall, Vancouver, BC V6T 1Z4 Canada; 2 Department of Emergency Medicine, University of Washington, Seattle, USA

**Keywords:** Biomaterials, Materials science, Biophysics, Biopolymers in vivo

## Abstract

The adhesion of blood clots to wounds is necessary to seal injured vasculature and achieve hemostasis. However, it has not been specifically tested if adhesive failure of clots is a major contributor to rebleeding and what mechanisms prevent clot delamination. Here, we quantified the contribution of adhesive and cohesive failure to rebleeding in a rat model of femoral artery injury, and identified mechanisms that contribute to the adhesive strength of bulk clots in a lap-shear test in vitro. In the rat bleeding model, the frequency of clot failures correlated positively with blood loss (R = 0.81, *p* = 0.014) and negatively with survival time (R =  − 0.89, *p* = 0.0030), with adhesive failures accounting for 51 ± 14% of rebleeds. In vitro, adhesion depended on fibrinogen and coagulation factor XIII (FXIII), and supraphysiological FXIII improved adhesive strength. Furthermore, when exogenous FXIII was topically applied into the wound pocket of rats, eleven adhesive failures occurred between eight rats, compared to seventeen adhesive failures between eight untreated rats, whereas the number of cohesive failures remained the same at sixteen in both groups. In conclusion, rebleeding from both adhesive and cohesive failure of clots decreases survival from hemorrhage in vivo. Both endogenous and exogenous FXIII improves the adhesive strength of clots.

## Introduction

Blood clots, like all sealants, fail either by cohesive failure or adhesive failure^1^. During cohesive failure, the sealant fractures internally from forces that disrupt structural integrity within the clot. Whereas during adhesive failure, the sealant remains mechanically intact, but its substrate interface is disrupted. It is crucial to understand the mechanisms behind the failure of blood clots as it may inform approaches to mitigate rebleeding during severe hemorrhage, but it is not known if adhesive failure contributes to hemostasis and rebleeding in vivo.

Blood coagulation is typically effective at sealing damaged blood vessels to achieve hemostasis, but blood clots can fail during severe hemorrhage, leading to rebleeding and secondary hemorrhage. Rebleeding is common during severe hemorrhage, normally occurring many times before definitive hemostasis is reached^2^. Rebleeding typically occurs when blood pressure exceeds the mechanical stability of the clot. This can occur when blood pressure increases during fluid resuscitation^2^. The failure of clots and volume of blood loss from rebleeds can be exacerbated by trauma-induced coagulopathies, including hyperfibrinolysis and the depletion of coagulation factors^3^, as well as physical perturbation of the clot during patient transport^4^. The destabilization of blood clots and subsequent rebleeding correlates with poor survival in trauma patients^2^. The critical stress a clot can withstand before internal fracture, its cohesive strength, is positively correlated with its elastic modulus, a measure of its stiffness, as measured by a clinically-utilized technique, thrombelastography (TEG)^5^. While the factors that contribute to clot stiffness have been extensively characterized, such as by using TEG^6,7^, the adhesive properties and mechanisms of clots, examined in bulk rather than examining individual components in isolation, are less characterized.

Several components of blood are known to have adhesive properties. Fibrin and coagulation factor XIII (FXIII) have been used as surgical sealants^8^. Fibrin is formed by proteolytic cleavage of fibrinopeptides of its precursor, fibrinogen, during activation of the coagulation cascade. The fibrin monomers then self-assemble to protofibrils, which aggregate into a mesh-like structure^9,10^. This fibrin polymer can both directly crosslink and mechanically interlock with extracellular matrix (ECM) proteins in tissue. FXIII is present in plasma as a heterotetrameric proenzyme (FXIII-A_2_B_2_) and is converted to the activated transglutaminase (FXIII-A*, referred to here as FXIIIa) by thrombin in the presence of Ca^2+^. FXIIIa mediates the covalent crosslinking of fibrin to ECM proteins. FXIIIa also crosslinks fibrin to antifibrinolytic proteins, such as α2-antiplasmin^11^, as well as fibrin subunits to each other, which increases the density of the fibrin network^12^. Both of these processes stabilize the blood clot against premature fibrinolysis, and clinical observations indicate that a low FXIIIa concentration in blood is correlated with hyperfibrinolysis in trauma patients^13^. Platelets are adhesive to materials in the subendothelial space, including collagen and von Willebrand Factor (vWF), through receptors such as glycoprotein (GP) VI and the GPIb-V-IX complex^14,15^. While fibrin, FXIIIa and platelets all have adhesive properties, it is not known which of these components contribute to the adhesive strength of the bulk clot*.* We hypothesized that rebleeding can occur due to adhesive failure, and that supplemental FXIII could increase the adhesiveness of clots during bleeding. To confirm whether the delamination of clots from wound tissue leads to rebleeding, we analyzed the modes of clot failure in a rat femoral artery bleeding model. To test if depletion of FXIII and other clot components would decrease clot adhesiveness, we measured adhesive clot failure of depleted plasma ex vivo, and also tested if reintroducing exogenous FXIII would recover adhesion.

## Methods

### Use of experimental animals, and human participants

Experiments involving rat models were approved by the University of Washington Institutional Animal Care and Use Committee (IACUC). All experiments were performed in accordance with IACUC guidelines and regulations.

Experiments involving human participants were approved by the Human Research Ethics boards of the University of British Columbia (UBC) and the Canadian Blood Services (CBS). All experiments were performed in accordance with UBC and CBS guidelines and regulations. Informed consent was obtained from all healthy human volunteers prior to whole blood donation.

All donors were deidentified. The recruitment criteria were inclusive of both sexes.

### Rat femoral artery injury model

Test reagents were applied to a rat model of femoral artery injury to evaluate their effects on clot failure mode and hemostatic efficacy. The procedure for the rat model was modified from a previous study^16^. Wild-type male Sprague–Dawley rats weighing between 300–450 g were used. Rats of only one sex were used to reduce sex-related variability in this pilot study, but should be extended in the future to involve both sexes. The rats were anesthetized with ketamine and xylazine and maintained under isoflurane for the duration of the protocol (roughly 1 h of pre-injury preparation as described below, 5 min of free bleeding, followed by 75 min of fluid resuscitation). Before injury, the carotid artery and jugular vein were catheterized to monitor blood pressure (BP) and to infuse saline, respectively. A tracheotomy was performed and a small polyethylene tube was inserted to aid respiration using a mechanical ventilator. The left femoral artery was isolated and clamped proximally and distally. A wound pocket of roughly 2 mL in volume was made at the incision site to access the femoral artery prior to isolation and incision of the artery to induce bleeding. A 3-mm longitudinal incision was then made in the anterior wall of the femoral artery segment between the clamps to allow for free bleeding. Prior to releasing the clamps, blood was drawn from the carotid artery through the inserted catheter to standardize starting blood pressure to as close to 50 mmHg as possible (no lower than 25, and no higher than 75 mmHg) to prevent exsanguination and standardize the initial bleed volume. Following catheter hemorrhage, clamps were removed to initiate bleeding from the wound, and this time was designated t = 0 min. Solutions containing the test reagents (200 µL of 0.9% saline for the control group, or 0.67 mg/mL FXIII for the treatment group) were applied immediately into the wound pocket and allowed to mix with the forming blood clot while still in its liquid phase. This FXIII concentration was chosen to correspond with a concentration that improved clot adhesion in vitro, accounting for the volume of the wound pocket and potential loss of the exogenous FXIII from the wound pocket during the initial bleeding. After 5 min of free bleeding to allow clot formation, fluid resuscitation was initiated. Saline (0.9%) was infused intravenously at 3 mL/kg/min to raise mean arterial pressure to 60 mmHg as needed. The wound pocket was video recorded continuously starting at t = 0 min. Each video was viewed by two blinded observers to review each rebleeding event to determine whether the clot failed by adhesion (from the edges) or cohesion (not from the edge, but locations such as the middle of clot). Pre-weighed cotton gauze was used to collect blood spilling over the wound to track blood loss every 5 min. The blood loss attributed to each clot failure event was estimated as the blood loss within the 5-min interval in which the failure occurred. When more than one failure occurred in the 5-min interval, blood loss was divided equally among the failures. Survivors were euthanized under anesthesia if surviving up to 80 min or if arterial wave form was lost and mean arterial pressure confirmed to be < 20 mmHg, whichever occurred first. Animals that did not survive the initial catheter hemorrhage were excluded from the analysis.

### Isolating red blood cells (RBC) and platelets from whole blood and reconstituting them in plasma

RBCs were obtained from human whole blood collected into Vacutainer tubes containing sodium citrate (0.105 M) (BD Biosciences). The blood was centrifuged at 100×*g* for 20 min to separate the RBC from platelet-rich plasma (PRP). The RBC fraction was centrifuged at a higher speed of 500 g for 5 min to separate residual plasma from the RBC. The RBC fraction was then washed twice with phosphate buffered saline (Gibco PBS Buffer, pH 7.4, Thermo-Fisher). In experiments that investigated the effect of RBCs on clot adhesion (Fig. 2a, b), the washed RBCs or a vehicle control were reconstituted with the PRP fraction prior to mechanical testing.

Platelets were isolated from platelet-rich plasma (PRP) that was processed by the CBS from whole blood pooled from four deidentified female and male donors. The PRP was stored at 22˚C with mild shaking on a small platelet incubator and agitator (Boekel Scientific, USA) and used within five days of the blood draw. To extract platelets, the PRP was centrifuged at 250 g for 20 min to pellet the platelets. The platelet-poor plasma (PPP) was removed from the pellet and the pellet was resuspended in citrate glucose saline buffer (120 mM NaCl, 30 mM D-glucose, 11 mM trisodium citrate, pH 6.5). The platelets were again centrifuged at 250 g for 10 min, the buffer removed, and the platelets resuspended in Tyrode’s HEPES buffer (1.8 mM CaCl_2_, 1.1 mM MgCl_2_, 2.7 mM KCl, 137 mM NaCl, 0.4 mM NaH_2_PO_4_, 10 mM HEPES, 5.6 mM D-glucose, pH 6.5) to prevent platelet activation prior to the start of the experiment. The platelets were centrifuged at 250 g for 10 min for a final wash. The buffer was removed and the platelets resuspended in fresh Tyrode’s HEPES and counted by a XN-550 Automated Hematology Analyzer (Sysmex). Platelet concentration was adjusted by the addition of more Tyrode’s HEPES as specified in the protocol for each experiment in Supplementary Table S1.

### Measuring the adhesion of blood clots using a double lap-shear test

Blood samples containing clotting reagents were prepared according to the recipes listed in Supplementary Table S1, with a total volume of 50 µL. Suppliers for clotting reagents are listed in Supplementary Table S2, and compositions of commercially-purchased human plasma products are listed in Supplementary Table S3. Normal pooled plasma was purchased from Affinity Biologicals, and was collected from over 20 deidentified female and male donors. Adhesion was measured with a TA Q800 Dynamic Mechanical Analyzer (DMA) equipped with a shear-sandwich clamp (TA Instruments). Collagen-coated glass coverslips (Corning, all experiments except Fig. 3f) or other types of glass coverslips (Neuvitro, uncoated or coated with collagen, fibronectin, or laminin; see Fig. 3f for details) were attached to the four clamp faces using UV-cured glue (Loon Outdoors). The blood samples were loaded and sandwiched between two coverslips that were separated by 0.25 mm. The blood sample was contained in the 10 × 10 mm section of the coverslip using fluorocarbon grease (Krytox grease, DuPont) to prevent evaporation (see schematic in Supplementary Figure S1). Clots were formed for 60 min at 37˚C unless otherwise specified. A force ramp was applied at 0.01 N/min by the DMA until the clots failed. Adhesive failure was confirmed by visual inspection that each of the two clots remained intact and had detached from one coverslip while remaining adhered to the other.

### Measuring clot stiffness and lysis

A Haemonetics TEG5000 thrombelastograph was used to measure the elastic modulus of the clot as it formed. The blood sample was 360 µL in volume, prepared according to the recipes listed in Supplementary Table S1.

Lysis was measured by transmission spectrometry. Plasma samples (100 µL) with or without tissue plasminogen activator (tPA) (257 ng/mL, Genentech) were added to a 96-well microplate using the recipes listed in Supplementary Table S1. The plate was read by a GENios microplate reader (Tecan) at 405 nm (A_405_) for 103 min. The A_405_ increased with increased turbidity as the clot became insoluble through fibrin formation and then decreased as the clot was lysed^17^. Clots were measured until complete lysis, defined by when the absorbance of all reactions had reached the baseline level as indicated by the no-calcium negative control.

### Statistical analysis

Normality was assumed for all data sets but could not be verified due to low sample sizes. A Pearson (R) calculator was used to calculate the Pearson’s correlation coefficient and p value for correlation analyses. One- and two-way analyses of variance (ANOVA) and the two-stage Benjamini, Krieger, and Yekutieli procedure, with adjusted p values for multiple comparisons, were used to calculate the p values between multiple groups. A Student’s *t* test was used to calculate the p values in specific comparisons between two groups of interest. SEM denotes standard error of mean, and is defined as the standard deviation of the data set divided by the square root of the number of replicate values recorded.

## Results

### Clot failures lead to increased blood loss and decreased survival.

**Figure 1:**
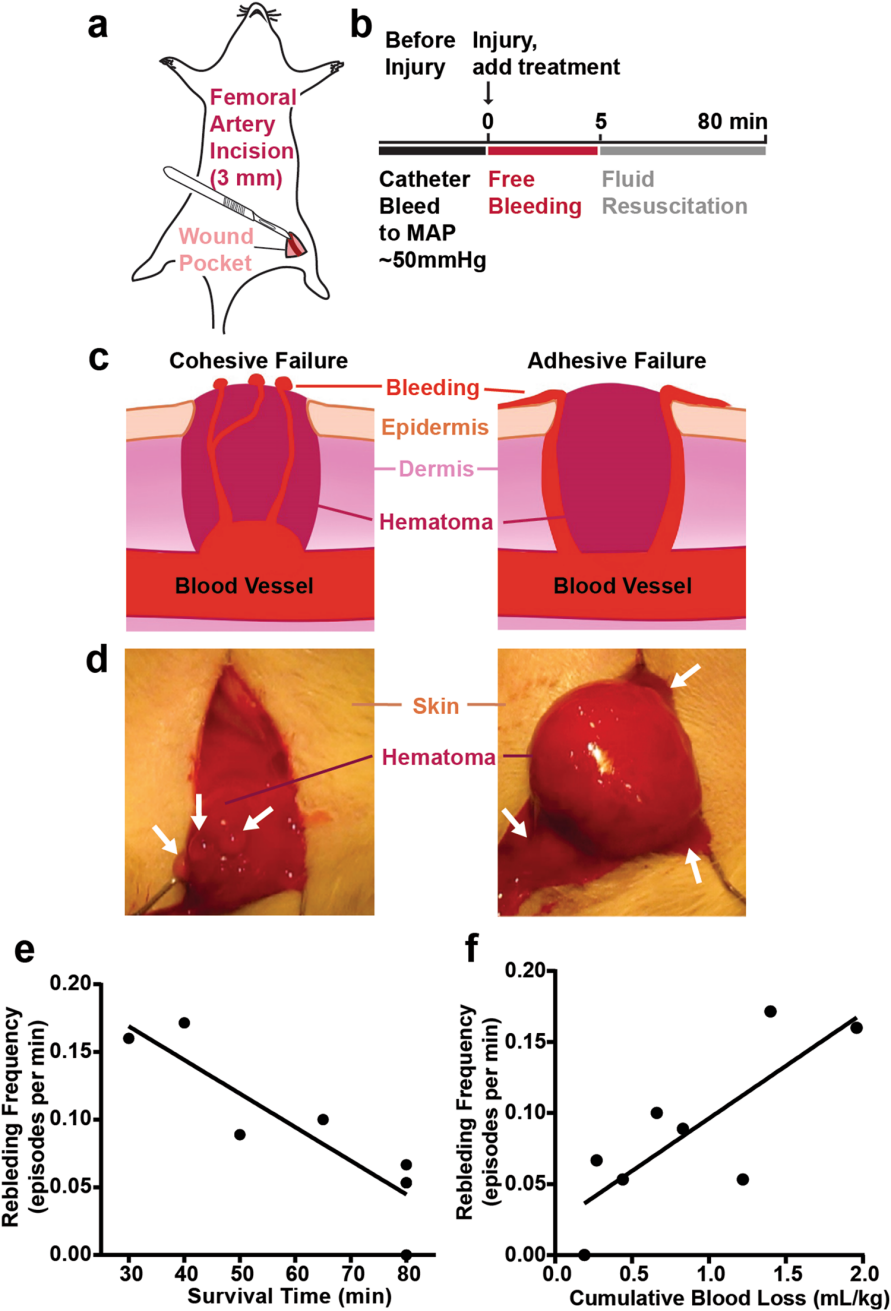
The failure of clots correlated with decreased survival time and increased blood loss in rats subjected to femoral artery incision followed by intravenous fluids to maintain blood pressure. (**a**) Schematic of rat injury. The femoral artery (dark red) was exposed and incised 3 mm longitudinally to induce bleeding. Blood was allowed to pool in the wound pocket (pink). (**b**) Timeline for the rat surgery performed. Before injury, blood was drawn from a catheter in the carotid artery to normalize mean arterial pressure (MAP) to approximately 50 mmHg. After the injury, treatment was immediately added into the wound pocket. Free bleeding occurred for 5 min before fluid resuscitation was initiated. (**c**,**d**) Schematic (**c**) and photographs (**d**) of rebleeds from hematomas above an incised rat femoral artery. Both cohesive failure (left images) and adhesive failure (right images) occurred. White arrows indicate locations of clot failure and rebleeding. (**e**,**f**) The frequency of rebleeding in each untreated control animal is correlated with shorter survival time (e, R =  − 0.89, *p* = 0.0030), and greater blood loss (f, R = 0.81, *p* = 0.014).

To determine if rebleeding occurs in a rat model of lethal hemorrhage, intravenous fluids were administered following a femoral artery incision to challenge the hemostatic capacity of the formed clot. Blood loss was quantified every 5 min by weighing the bleed volume soaked by pre-weighed gauze (Fig. 1a,b). Failure mode of the hematoma was recorded, with “adhesive failure” denoting when the clot detached from the tissue to which it was attached, and “cohesive failure” denoting when rebleeding occurred in cracks within the clot itself (Fig. 1c,d). We found that adhesive failure was common, accounting for 51 ± 14% of the rebleeds between eight rats in this model. We found that clot formation and rebleeding events were common, with a mean of 4.1 and a standard deviation (SD) of 1.9 per rat. The frequency of rebleeds during fluid resuscitation correlated negatively with survival time (R = -0.89, p < 0.005, Fig. 1e), showing that animals with a higher number of clot failures had poorer survival. The frequency of rebleeds also correlated positively with cumulative blood loss (R = 0.81, p = 0.014, Fig. 1f).

### Red blood cells (RBC) and platelets have a minimal effect on bulk adhesion of clots.

**Figure 2:**
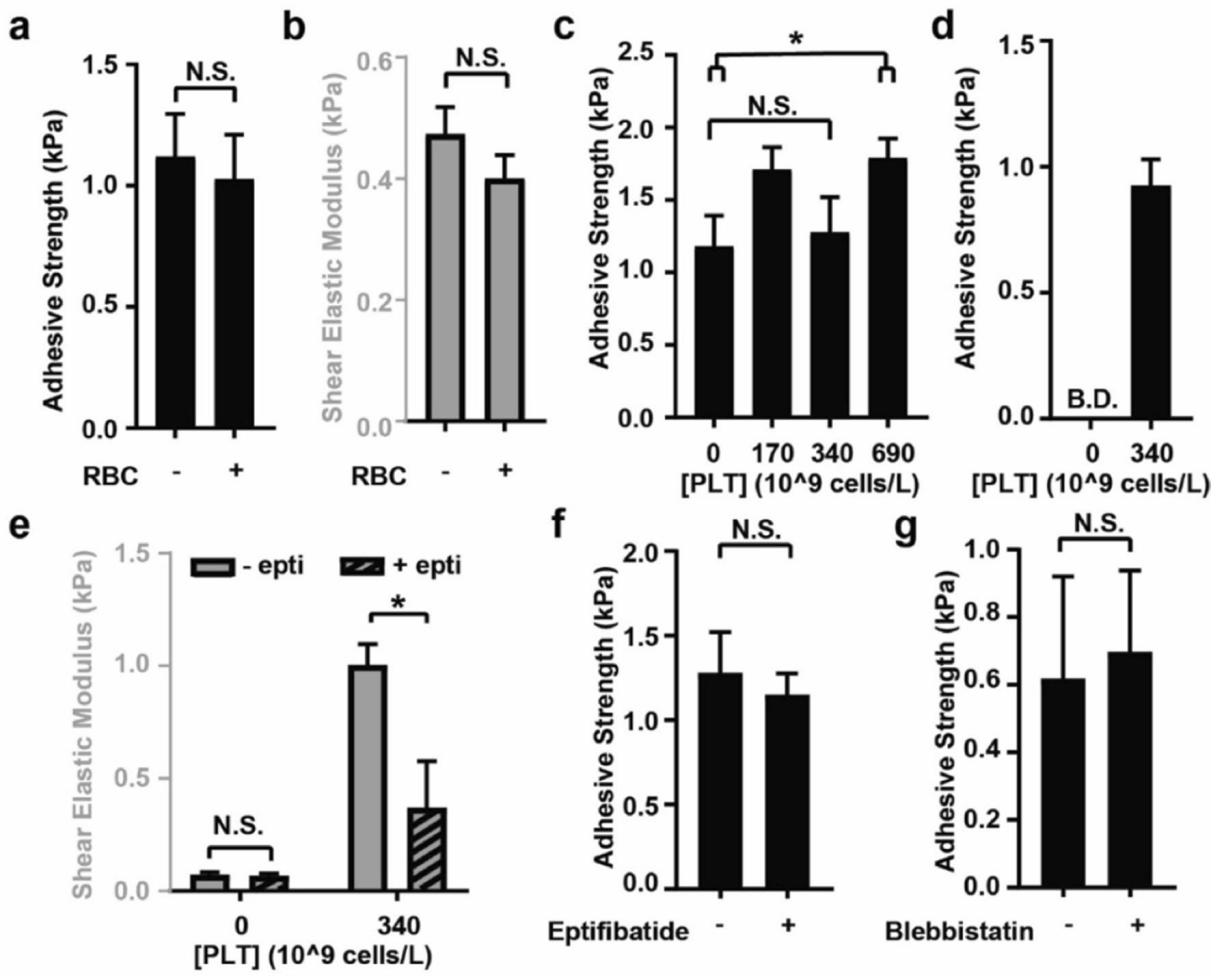
The effect of cellular components on the clot adhesive strength to collagen and the maximum clot stiffness. Black bars indicate the bulk clot adhesive strength to collagen measured by the lap-shear assay. Gray bars indicate the maximum clot stiffnesses (shear elastic modulus) measured by TEG. (**a**, **b**) RBC did not change clot adhesive strength (**a**) or stiffness (**b**). (**c**) The adhesive strength at various concentrations of platelets in a mature clot, measured 1 h after clotting initiated. (**d**) The adhesive strength of platelet-rich plasma at a normal physiological concentration (340 × 10^9^ cells/L), measured only 2 min after clotting was activated. (**e**) The clot stiffness of PRP with and without eptifibatide (epti) using TEG. Eptifibatide is an inhibitor of the platelet receptor GPIIb/IIIa, which is required for platelet aggregation and contraction. (**f**,**g**) The adhesive strength of PRP, measured 60 min after clotting was activated, was not changed by eptifibatide (**f**) or blebbistatin (**g**). Blebbistatin is an inhibitor of cytoskeletal actin polymerization, required for platelet adhesion, aggregation and contraction. Data bars indicate mean ± SEM, n = 5–8, *p < 0.05. N.S. denotes “not significant”. B.D. denotes “below detection”.

A lap-shear test was used to further investigate how the composition of blood clots affect its adhesive strength to collagen, a major component of wound tissues. The contributions of RBC to adhesive strength was first tested 1 h after the clots were formed. There was no significant difference in adhesive strength between PRP clots with or without RBC added (1.0 ± 0.2 and 1.1 ± 0.2 kPa respectively, Fig. 2a). This response was consistent with the effect that RBC have on clot stiffness (Fig. 2b)^18–20^. To test if platelets contribute to the adhesive strength of bulk clots to collagen in vitro, PRP clots were prepared by supplementing platelet-poor plasma (PPP, physiological concentration of fibrinogen) with washed platelets for final concentrations that mimic the low (170 × 10^9^ cells/L), normal (340 × 10^9^ cells/L) and high (690 × 10^9^ cells/L) physiological concentrations in blood. Adhesive strengths of PPP clots and PRP clots at low and normal platelet concentrations were not significantly different. At high platelet concentrations, PRP clots were 52% more adhesive than PPP clots (1.8 ± 0.1 and 1.2 ± 0.2 kPa respectively, Fig. 2c). At an earlier timepoint of 2 min after initiation of clot formation, PRP clots with normal platelet concentrations were adhesive, but PPP clots were not (0.9 ± 0.1 kPa vs. below detection, Fig. 2d). While the effects on adhesive strength were mild, platelets had a large effect on clot stiffness. PRP with a normal platelet concentration of 340 × 10^9^ cells/L had a 16-times greater stiffness than PPP (Fig. 2e). Treating the samples with inhibitors of platelet and clot contraction, eptifibatide (Fig. 2f) or blebbistatin (Fig. 2g), significantly reduced clot stiffness, but did not affect the adhesive strength of PRP clots.

### Fibrinogen and FXIII are major contributors to bulk clot adhesion

**Figure 3:**
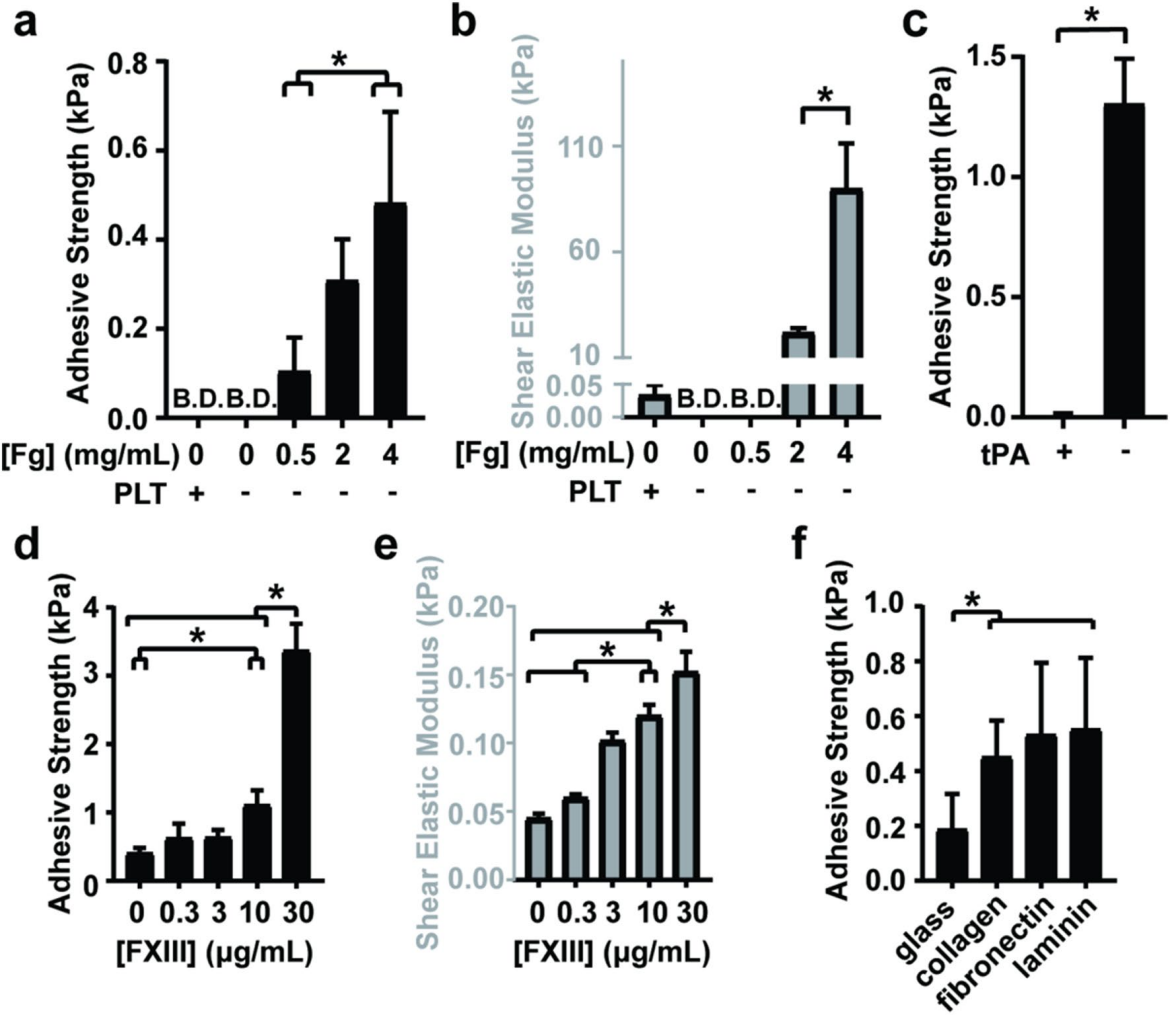
Fibrin, FXIII and ECM proteins enhance clot adhesion. Black bars indicate the bulk clot adhesive strength to collagen measured by the lap-shear assay and gray bars indicate the maximum clot stiffnesses (shear elastic modulus) measured by TEG. **(a**,**b)** Fibrin controlled clot adhesive strength (a) and clot stiffness (**b**) of both PPP and PRP clots. (**c**) Adhesive strength of clots treated with tPA is lost at 60 min. (**d,e**) FXIII increased both clot adhesive strength (**d**) and clot stiffness (**e**) of PPP clots. (**f**) The adhesion of erythrocyte-rich, platelet-rich whole blood clots to plain glass and glass coated with ECM proteins. Data bars indicate mean ± SEM, n = 3–7, *p < 0.05. B.D. denotes “below detection”.

To test the effects of fibrinogen (Fg) and FXIII on clot adhesion to collagen in vitro, clots were formed between collagen coverslips for 1 h and subjected to a lap-shear test until adhesive failure occurred. The concentrations of Fg were varied by supplementing purified Fg to Fg-poor PPP (PPP with less than 0.1 g/L fibrinogen). Supplementing with Fg increased the adhesive strength in a concentration-dependent manner (Fig. 3a). This response was consistent with the effect that Fg had on clot stiffness (Fig. 3b). Fg-poor PRP clots with platelets at approximately 690 × 10^9^ cells/L, were also not adhesive, and had minimal stiffness. Clots treated with tPA lost adhesive strength before 60 min (Fig. 3c). Lap shear tests performed at earlier timepoints showed that adhesive strength was lost at 46 min, before complete clot lysis was detected by spectrophotometry (Supplementary Figure S2). To test the effect of FXIII on the adhesion of bulk clots in vitro, varying concentrations of purified FXIII were supplemented to FXIII-poor PPP (PPP with less than 0.01 U/mL FXIII). Clot adhesive strength increased approximately 2-times at a physiological concentration of FXIII (10 µg/mL) compared to FXIII-poor PPP (1.1 ± 0.2 and 0.4 ± 0.1 kPa respectively). Increasing the FXIII concentration to 30 µg/mL led to a further 3-times increase of adhesive strength compared to the 10 µg/mL FXIII concentration (3.4 ± 0.4 kPa, Fig. 3d). Clot stiffness also increased by approximately 2-times from FXIII-poor PPP to 10 µg/mL FXIII, and an additional 27% when increased to a concentration of 30 µg/mL (Fig. 3e). Whole blood clots were more adhesive to extracellular matrix proteins collagen, fibronectin, and laminin, compared to uncoated glass (Fig. 3f).

### Topical supplementation of FXIII decreases incidences of adhesive failure

**Figure 4:**
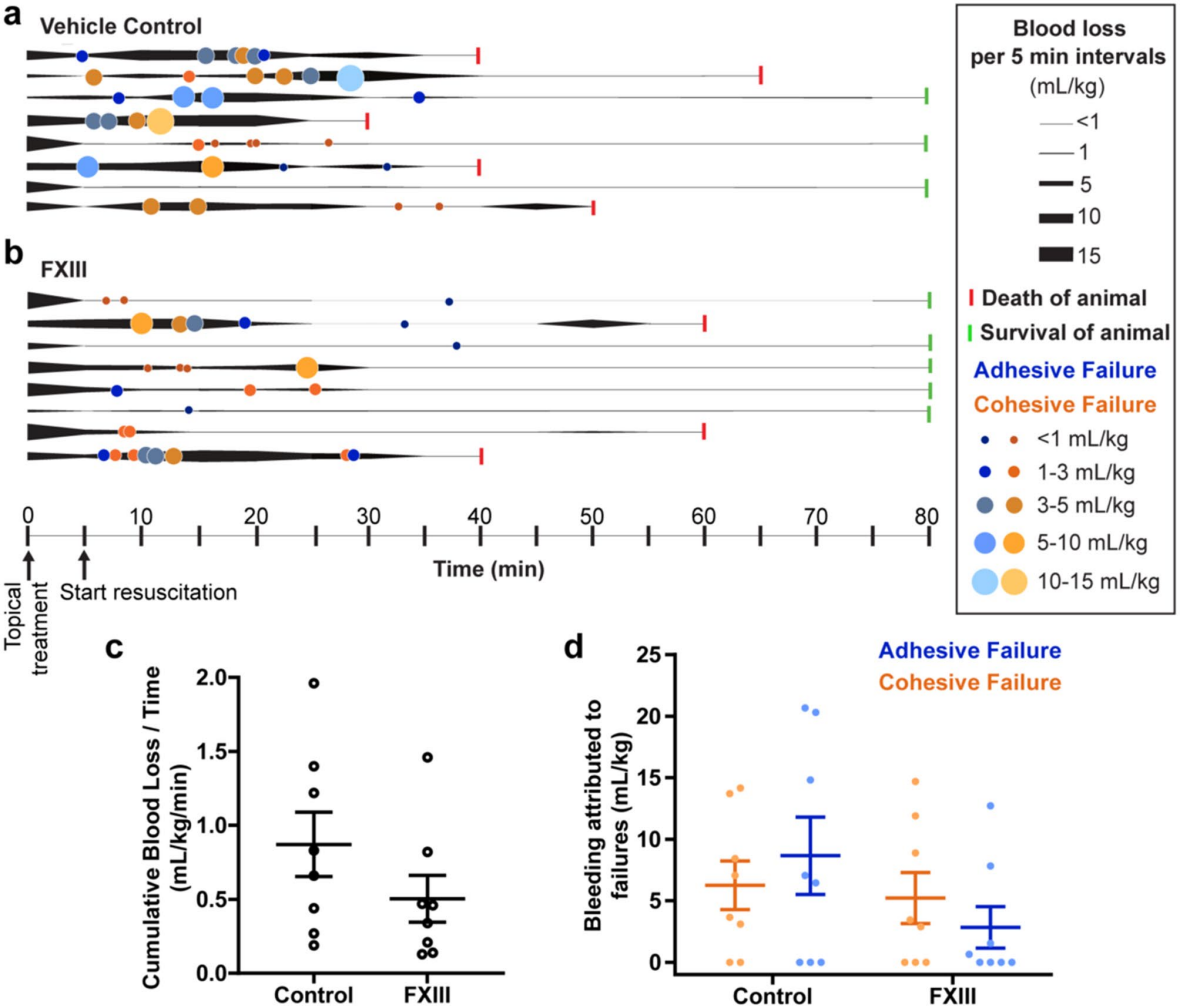
Rats with femoral artery hemorrhage experienced fewer severe adhesive failures when treated with topical FXIII. (**a**, **b**) The bleeding profiles of treated rats, where each line represents an individual animal within either the vehicle control (**a**) or topical FXIII-treated (**b**) experimental groups. The thickness of the black lines indicates the rate of blood loss, measured every 5 min. Circles indicate incidences of adhesive failure (blue) and cohesive failure (orange). The size of the circle indicates the severity of the immediate rebleeding following the clot failure. Vertical bars indicate death (red) or survival (green). Coloured bars indicate survival time of the animals. (**c**) Cumulative blood loss, showing the total hemorrhage volume for each animal at the end of the procedure divided by survival time. (**d**) Rebleeding attributed to each clot failure mode, estimated as the blood loss within the 5-min interval in which the failure occurred. Data bars indicate mean ± SEM, n = 8.

To test if adding topical FXIII to a wound would decrease blood loss from adhesive failures, 200 µL of a solution of FXIII (0.67 mg/mL) was added directly to the wound pocket of rats subjected to femoral artery puncture prior to fluid resuscitation (Fig. 4a,b). FXIII-A_2_B_2_ was chosen for this study, rather than FXIIIa, to ensure FXIIIa was only generated at the site of coagulation and minimize off-target crosslinking of proteins by purified FXIIIa. This treatment was compared to the vehicle control of 0.9% saline, using groups of eight rats. Blood in the systemic circulation of the rats was diluted by intravenous administration of saline, reaching approximately 53% and 55% of the original concentration after 20 min in the control and FXIII groups respectively (Supplementary Figure S3). In both the control and the FXIII-treated groups, a total of 16 cohesive failures occurred, while the number of adhesive failures decreased from 17 in the control group to 11 in the FXIII-treated group. The number of animals that survived the 80 min bleeding procedure was 3 of 8 control rats and 5 of 8 FXIII-treated rats. Cumulative blood loss divided by survival time was 0.9 ± 0.2 mL/kg/min for the control rats and 0.5 ± 0.2 mL/kg/min for FXIII-treated rats (p = 0.19 via Student’s *t* test, Fig. 4c). When blood losses from individual clot failures were added together, there was 8.7 ± 3.3 mL/kg of blood loss from adhesive failures in control rats and 2.8 ± 1.1 mL/kg in FXIII-treated rats (p = 0.13 via Student’s *t* test, Fig. 4d). Furthermore, no adhesive failures led to more than 5 mL/kg bleeding in the FXIII-treated rats whereas four such severe failures occurred in the group of control rats. While the study was not designed to measure the contraction of the clots in wounds, and the wounds were held open, we measured the surface area of the clots to detect any major difference in contraction. The change in surface area of the clots were similar between the groups, shrinking by 90 ± 3% in the FXIII-treated group compared to 91 ± 2% in the control group (p = 0.65, Supplementary Figure S4).

## Discussion

Blood clots seal injured vasculature, and thus clots must adhere to the wound site to achieve hemostasis. However, no studies had previously quantified adhesive failure in wounds and correlated them to survival, and it was not clear what mechanisms contribute to clot adhesive strength. This work shows for the first time that adhesive failures are common. We found that adhesive failures accounted for 51 ± 14% of rebleeds in untreated control animals. The in vitro results also highlight the important contribution that FXIII plays in preventing adhesive failure of the clot, presumably by enhancing the attachment of fibrin to ECM proteins.

The stiffness of blood clots as measured by thrombelastography correlates with clot cohesive strength and is used often to effectively predict the severity of bleeding^5^. Our studies that measured clot stiffness and lysis were specifically designed and performed in order to have direct comparisons to clot adhesion. Comparing the results from the lap-shear adhesion assay and the TEG assay showed that the stiffness of a clot was not directly correlated to its adhesive strength. For example, while platelets dramatically increased clot stiffness by approximately 16-fold, consistent with previous reports on the contribution of platelets to stiffness^21^, the clot adhesive strength to collagen was unchanged by platelets at the same concentration, and only increased by 1.5-fold at twice this concentration. This reflects the importance of platelets in clot cohesion through aggregation and binding of cells and proteins in blood^22^. While adhesives require cohesion to function, adhesive strength is also affected by the interactions between the adhesive and its substrate^23^. While platelets had only a mild effect on the adhesive strength of a formed clot 60 min after initiating clotting, they did contribute to adhesion at a very early stage of coagulation; PRP had higher adhesion than PPP at 2 min. This may have been due to platelets quickly binding to the adherend and enabling the initial attachment of blood components to collagen, adhering through receptors such as glycoprotein VI and Ia/IIa^24,25^. It is important to note that the adhesion test used here was a static system. In microfluidic studies, platelets are important in mediating the initial attachment and subsequent clot formation on collagen surfaces under flow^26^, and platelets may have a greater impact on mature clots under flow. RBC count did not affect the clot adhesive strength. This was similar to the effect of RBC on clot stiffness. It is known that hematocrit levels do not improve clot stiffness as measured by thromboelastrography^18–20^. This could be due to the deformability of RBC^27^. In conditions where RBC become less deformable, such as sickle cell disease, clot stiffness increases with RBC concentration^28^.

We observed that clot adhesive strength can be affected by both its intrinsic composition and the interactions between the clot and the substrate to which it is adhered. Both fibrin and FXIII improved clot adhesion to collagen, in the absence of platelets, in a dose dependent manner. This suggests that perhaps the primary contributions of fibrin and FXIII on enhancing clot attachment to the collagen surface was independent from the known role of platelets in mediating fibrin-collagen interactions. Unsurprisingly, fibrin was essential for clot stiffness^29,30^. Weak clot adhesion occurred in PPP deficient in FXIII, but supplementing FXIII increased the clot’s adhesive strength. In particular, a high dose of FXIII (30 µg/mL) increased clot adhesive strength by roughly 3-times compared to a lower physiological concentration (10 µg/mL). Consistent with previous reports^31^, clot stiffness also increased by adding FXIII, but the high dose of FXIII only increased clot stiffness moderately (27%) compared to the lower physiological concentration tested. We found that concentrations of FXIII that achieved only modest increases in clot stiffness led to large increases in adhesive strength. This highlights that in addition to increasing clot cohesion through interchain crosslinking of fibrin strands, FXIIIa plays an important role in enhancing the attachment of fibrin to wound tissue. Whole blood clots were more adhesive to extracellular matrix proteins, collagen, fibronectin, and laminin, compared to plain glass, indicating that both the blood composition and the substrate to which the clot is adhered affect clot adhesion.

After showing in vitro that supplementing FXIII enhanced clot adhesion, we tested if supplementing FXIII directly into the wound site would prevent adhesive failure in our rat hemorrhage model. Of rats that received FXIII, more survived, fewer adhesive failures and lower blood loss occurred compared to control animals. While these comparisons between the control and FXIII-treated groups of rats were not statistically significant with this sample size of eight rats per group, these in vivo results warrant further investigations into the effects of FXIII on clot adhesive strength in future studies with a larger sample size. Models of hemorrhage typically have high variability in survival rate and blood loss. The model used in this study also showed high variability in the mode of clot failures. A larger sample size would be needed to better distinguish the effects of exogenous treatments from intrinsic differences between animals and injuries. Future studies using rats with a genetic knockout of FXIII would also be useful for testing the physiological contribution of FXIII^32^.

It is probable that clot contraction influences adhesion of clots, but this was not specifically tested in this study. Inhibitors of clot contraction, eptifibatide and blebbistatin, did not influence adhesion in the specific in vitro assay used here. In vivo, some shrinkage of the clots occurred even though the wounds were held open, although we did not detect differences in clot contraction between the treatment groups.

In conclusion, the bulk adhesion of clots contributes to hemostasis in vivo. Clot failures were associated with blood loss and poor survival, and adhesive and cohesive failures were equally common. Bulk clot adhesion is controlled by fibrin and FXIII, but only minimally by platelets in vitro, demonstrating that clot stiffness and clot adhesion do not have a linear relationship. This highlights the importance of mechanisms of clot adhesion to hemostasis and encourages the development of therapeutics for bleeding disorders or hemorrhage that enhance these innate mechanisms.

## Supplementary information

Supplementary Information.
