# Supplementary material for: The adhesion of clots in wounds contributes to hemostasis and can be enhanced by coagulation factor XIII
Source: Sci Rep. 2020 Nov 18;10:20116. doi: 10.1038/s41598-020-76782-z (PMC7675984; doi:10.1038/s41598-020-76782-z)
Supplement: Supplementary file 1 — Supplementary Information. [file 41598_2020_76782_MOESM1_ESM.docx]

**Supplementary information for: “The Adhesion of Clots in Wounds Contributes to Hemostasis and can be Enhanced by Coagulation Factor XIII”**

Karen Y.T. Chan, Alyssa S. M. Yong, Xu Wang, Kristyn M. Ringgold, Alexander E. St. John, James R. Baylis, Nathan J. White, Christian J. Kastrup


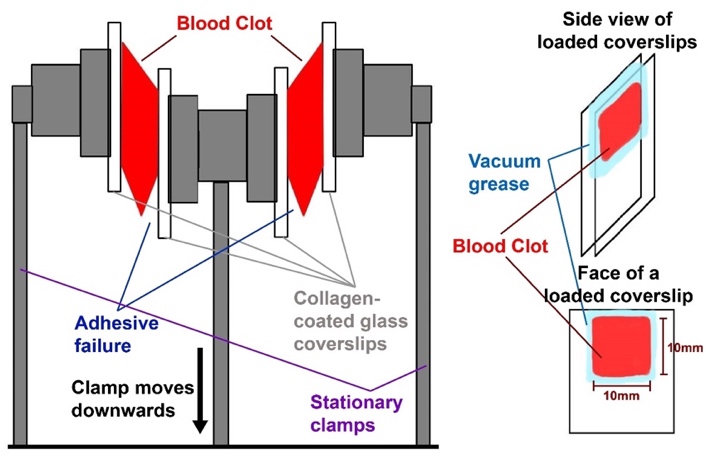


**Supplementary Figure S1. Schematic of lap-shear adhesive strength test.** A double lap-shear apparatus was used, where the middle clamp was moved downwards to deliver shearing force on the samples. Blood samples were loaded into vacuum grease-lined wells between collagen-coated glass coverslip pairs. The grease was used to prevent evaporation of samples, and the small force generated by the grease was subtracted from the adhesion measurement.


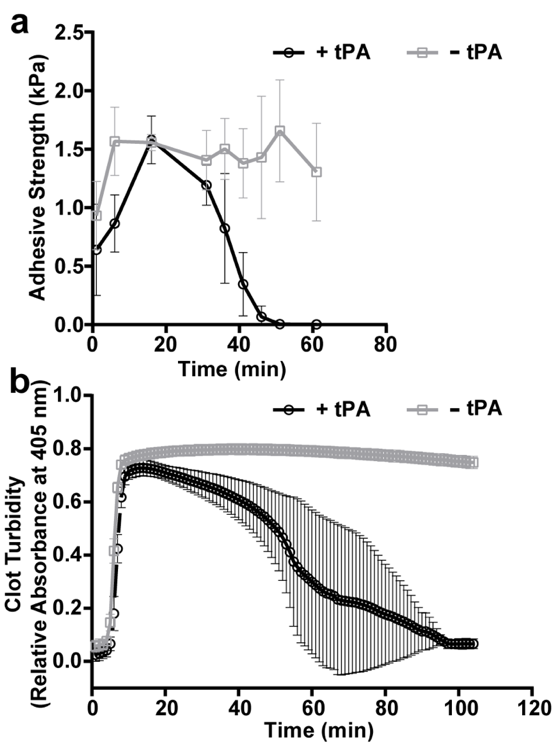


**Supplementary Figure S2. Clots lose adhesion before full clot lysis during tPA-induced fibrinolysis.** **a)** The adhesive strength of PPP clots with and without tPA. **b)** Fibrinolysis of bulk PPP clots with and without tPA, measuring clot turbidity by spectrophotometry. Data bars indicate mean ± SEM, n = 3-5.

**
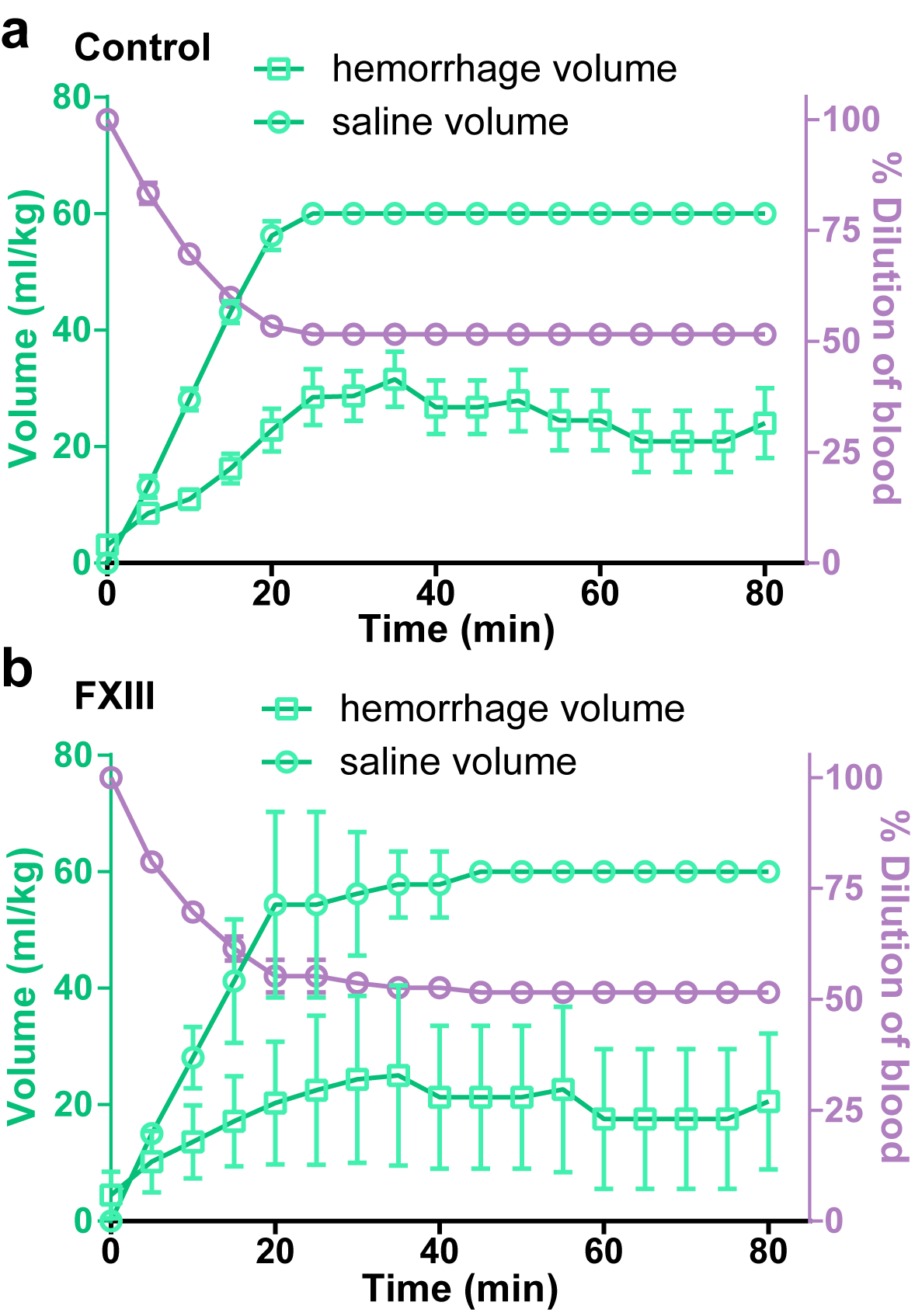
**

**Supplementary Figure S3.** **Blood loss and fluid resuscitation and the resulting estimated dilution of blood in rats following femoral artery puncture.** The extent of blood dilution is shown for rats **a)** without topical treatment, and **b)** treated topically with FXIII.


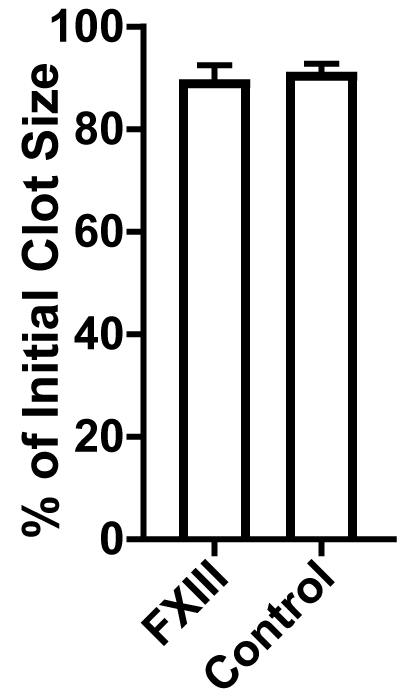


**Supplementary Figure S4. Shrinkage in the surface area of clots during femoral artery hemorrhage in rats.** Rats were either administered topical saline (vehicle control), or FXIII.

| Figure number | Hematocrit (% total volume) | Platelet (10^9^ cells/L) | Plasma (% total volume)^1^ | CaCl_2_ (mM) | Innovin (% total volume)^2^ | Other modulators |
| --- | --- | --- | --- | --- | --- | --- |
| 2a,b | 45 | Variable^3^ | 51 (donor) | 15 | 2 | None |
| 2c-g | 0 | 343, unless otherwise indicated on the figure | 44 | 15 | 2 | Eptifibatide (160 µM), Blebbistatin (300 µM, in 3.2% DMSO) |
| 3a,b | 0 | 0 or 690, indicated on the figure | 56 | 15 | 2 | Fibrinogen varied, see figure |
| 3c, S2 | 0 | 0 | 94 | 15 | 2 | tPA (256 ng/mL) |
| 3d,e | 0 | 0 | 92 | 15 | 2 | FXIII varied, see figure |
| 3f | Fresh donor whole blood used at 96% v/v | | | 15 | 2 | None |

**Supplementary Table S1. Reaction mix recipes for lap-shear adhesive strength test, thromboelastography, and spectrophotometric clot lysis assay.**

Platelets were isolated from pooled platelet-rich plasma (PRP) that was processed by the Canadian Blood Services from whole blood pooled from four deidentified female and male donors.
2 Innovin molar concentrations not declared by manufacturer (Dade Behring, Marburg, Germany)

3 Platelet counts variable between donors and not normalized. PRP removed by centrifugation and reconstituted with or without RBC

| Material | Figure | Supplier | Catalogue number |
| --- | --- | --- | --- |
| Dade Innovin Reagent | 2,3 | Siemens Healthineers AG (Erlangen, Germany) | 10873566 |
| Human normal control plasma | 2c-g, 3c, S2 | Affinity Biologicals Inc. (Ancaster, Canada) | UFNCP0125 |
| Eptifibatide | 2e,f | MilliporeSigma Canada Co. (Oakville, Canada) | SML1042 |
| Blebbistatin | 2g | MilliporeSigma Canada Co. (Oakville, Canada) | B0560 |
| Human fibrinogen-deficient plasma (defibrinated through using human thrombin) | 3a,b | Affinity Biologicals Inc. (Ancaster, Canada) | FG-DP |
| Human fibrinogen | 3a,b | Haematologic Technologies Inc. (Essex Junction, USA) | HCI-0150R |
| Human FXIII-deficient plasma (depleted using FXIII antibodies) | 3d,e | Affinity Biologicals Inc. (Ancaster, Canada) | FXIII-DP |
| Human FXIII (zymogen, contains A_2_B_2_ subunits) | 3d,e | Haematologic Technologies Inc. (Essex Junction, USA) | HCXIII-0160 |
| Human FXIII (zymogen, contains A_2_B_2_ subunits) | 4b-d | Enzyme Research Laboratories (South Bend, USA) | HFXIII 1313 |

**Supplementary Table S2. Suppliers for materials used in *in vitro* assays.**

| Plasma | Figure | FXIII (activity) | Fibrinogen (g/L) |
| --- | --- | --- | --- |
| Human fibrinogen-deficient plasma | 3a,b | Unassayed | 0.049 |
| Human FXIII-deficient plasma | 3c,d | <0.01 U/mL | 2.71 |

**Supplementary Table S3. FXIII activity and fibrinogen concentration of deficient plasmas used in *in vitro* assays.**
